# Berberine Activates Aryl Hydrocarbon Receptor but Suppresses CYP1A1 Induction through miR-21-3p Stimulation in MCF-7 Breast Cancer Cells

**DOI:** 10.3390/molecules22111847

**Published:** 2017-10-28

**Authors:** Sheng-Nan Lo, Chun-Wei Wang, Yueh-Shieh Chen, Chiung-Chiao Huang, Tian-Shung Wu, Lih-Ann Li, I-Jung Lee, Yune-Fang Ueng

**Affiliations:** 1 Division of Basic Chinese Medicine, National Research Institute of Chinese Medicine, Taipei 112, Taiwan; loshengnan@gmail.com (S.-N.L.); bubblecod@hotmail.com (C.-W.W.); kari0734@nricm.edu.tw (Y.-S.C.); josephine797@gmail.com (C.-C.H.); 2 Institute of Biopharmaceutical Sciences, School of Pharmacy, National Yang-Ming University, Taipei 112, Taiwan; 3 Department of Chemistry, National Cheng-Kung University, Tainen 701, Taiwan; tswu@mail.ncku.edu.tw; 4 National Institute of Environmental Health Sciences, National Health Research Institutes, Zhunan, Miaoli 350, Taiwan; lihann@nhri.org.tw; 5 Division of Chinese Materia Medica Development, National Research Institute of Chinese Medicine, Taipei 112, Taiwan; lee@nricm.edu.tw; 6 Institute of Medical Sciences, Taipei Medical University, Taipei 101, Taiwan; 7 Department and Institute of Pharmacology, School of Medicine, National Yang-Ming University, Taipei 112, Taiwan

**Keywords:** berberine, aryl hydrocarbon receptor, CYP1A1, miR-21-3p, breast cancer cells

## Abstract

Berberine and the methylenedioxy ring-opening derivatives palmatine and jatrorrhizine are active ingredients in immunomodulatory plants, such as goldenseal. This study aimed to illustrate the effects of protoberberines on aryl hydrocarbon receptor (AhR) activation and cytochrome P450 (CYP) 1 in the estrogen receptor (ER)α(+) MCF-7 breast cancer cells. Among protoberberines at non-cytotoxic concentrations (≤10 μM), berberine had the most potent and statistically significant effects on AhR activation and CYP1A1/1A2/1B1 mRNA induction. The 24-h exposure to 10 μM berberine did not change CYP1A1 mRNA stability, protein level and function. Berberine significantly increased micro RNA (miR)-21-3p by 36% and the transfection of an inhibitor of miR-21-3p restored the induction of CYP1A1 protein with a 50% increase. These findings demonstrate that the ring opening of the methylenedioxyl moiety in berberine decreased AhR activation in MCF-7 cells. While CYP1A1 mRNA was elevated, berberine-induced miR-21-3p suppressed the increase of functional CYP1A1 protein expression.

## 1. Introduction

The aryl hydrocarbon receptor (AhR) downstream targets, cytochrome P450 (CYP) 1 isoforms, play very important roles in the detoxification and bioactivation of environmental pollutants, carcinogens and physiological compounds, such as benzo(a)pyrene (B(a)P) and estradiol (E2) [1]. In the determination of CYP1 function, 7-ethoxyresorufin is generally used as a common substrate of CYP1s, and CYP1A1 has the highest turnover rate in 7-ethoxyresorufin *O*-deethylation (EROD) [2]. CYP1A1 and CYP1B1 are predominantly expressed in extrahepatic tissues including the breasts (mammaries) [3]. Although CYP1A2 is predominantly a hepatic isoform, CYP1A2 protein has been identified in breast cancer tissues [4]. 2,3,7,8-Tetrachlorodibenzo-*p*-dioxin (TCDD) and B(a)P induced CYP1 through the binding of the AhR [5]. The AhR nuclear translocator (ARNT) carries the AhR–ligand complex to interact with the dioxin-responsive element (DRE) of the *CYP1A1*, *CYP1A2* and *CYP1B1* genes, resulting in transcriptional induction. Compared to the dramatic increase in CYP1A1 mRNA by TCDD in estrogen receptor (ER)α(+) MCF-7 (human breast adenocarcinoma) cells [6], ERα(-)MDA-MB-231 (MB-231) (human breast adenocarcinoma) cells carry an ARNT variant with an incomplete glutamine-rich region and transactivation domain, resulting in an inefficient CYP1A1 induction [7]. In addition, the cross-talk between ERα and AhR can affect CYP1A regulation. The TCDD- and β-naphthoflavone-induced levels of CYP1A1 mRNA were reduced in the ERα knockdown mouse mammary epithelial cell line HC11 (nontumorigenic) and the liver of ovariectomized ER knockout mice, respectively [8]. However, in the MCF-7 cell line, TCDD-induced CYP1A1 mRNA levels were not affected by ERα knockdown. In MCF-7 cells, concurrent exposure to E2 reduced the TCDD-mediated induction of CYP1A1 mRNA through the ERα-dependent increase of DNA methylation of DRE [9]. The AhR activation and E2 are important factors in the regulation of mammary CYP1s.

**Scheme 1:**
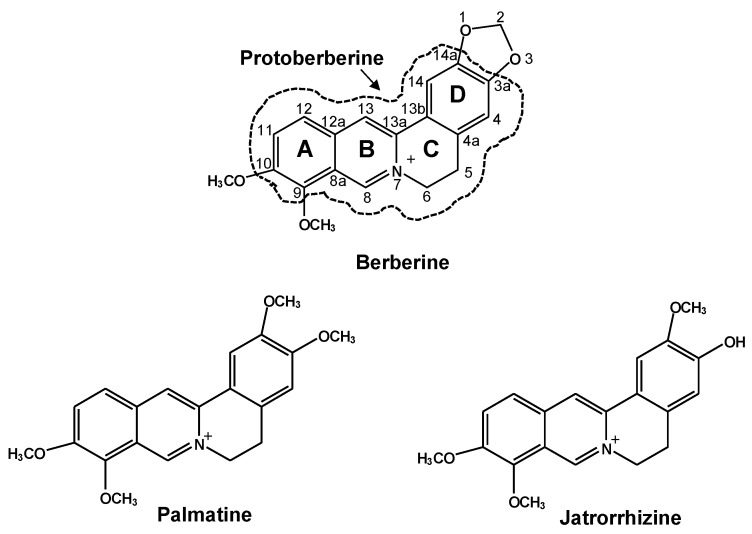
The chemical structures of the protoberberines berberine, palmatine and jatrorrhizine. The area within the dashed circle shows the core structure of the protoberberine.

The structurally diverse protoberberines, berberine, palmatine and jatrorrhizine (Scheme 1), are active ingredients in the immunomodulatory medicinal plants *Coptis chinesis* (huanglian) and *Hydrastis canadensis* (goldenseal) and are also candidates for breast cancer therapy [10,11]. Our previous report demonstrated that these three protoberberines could inhibit recombinant human CYP1A1 activity [12]. A CYP1A1 inhibitor can activate AhR indirectly through inhibiting the oxidation of a photoproduct of tryptophan, 6-formylindolo(3,2-*b*)carbazole, in cultured cells [13]. In an AhR-mediated DRE activation system, the activation fold of reporter activity by berberine (50 μM) was about 30% of that of TCDD (5 nM) in a rat hepatoma cell line H4IIE [14]. Berberine could not compete with TCDD for the binding to AhR in mouse hepatoma cells Hepa-1c1c7, suggesting that berberine could be a weak ligand or might activate AhR indirectly. Palmatine (≥10 μM) activated DRE and increased CYP1A1 mRNA and EROD activity in HepG2 cells, whereas EROD activity remained the same in the primary culture of human hepatocytes [15]. The modulation of CYP1s by protoberberines showed dependence on cell types and the effect of palmatine and jatrorrhizine was not explored in breast cancer cells.

In mice, berberine (300 mg/kg) increased hepatic Cyp1a2 mRNA without affecting EROD activity [16]. In HepG2 cells, berberine elevated CYP1A1 mRNA until it approached TCDD-induced levels [14]. However, the increase of CYP1A1 protein by berberine was only ~16% of that by TCDD, suggesting that berberine might cause post-transcriptional down-regulation of CYP1A1 expression. By using univariate analysis, human hepatic cytochrome P450 (P450, CYP) 1A1 protein levels were negatively correlated with miR-21-5p (mature miR-21), miR-132 and miR-142-3p levels [17]. In HepG2 cells, berberine induced the miR-21 passenger strand miR-21-3p (miR-21*), resulting in decreased methionine adenosyltransferases and apoptosis [18]. The influence of miR-21-3p on CYP1A1 expression remained unclear. This is the first report to reveal the differential effects of palmatine and jatrorrhizine at non-cytotoxic concentrations on AhR activation and CYP1 regulation in breast cancer cells. The effects of E2, ERα knockdown and miRs on the berberine-mediated CYP1A1 regulation is reported for the first time. For the purpose of comparison, MB-231 cells were studied as the constitutively inefficient AhR- and ERα-bearing cells.

## 2. Results

### 2.1. DRE Activation by Berberine, Palmatine and Jatrorrhizine

**Figure 1:**
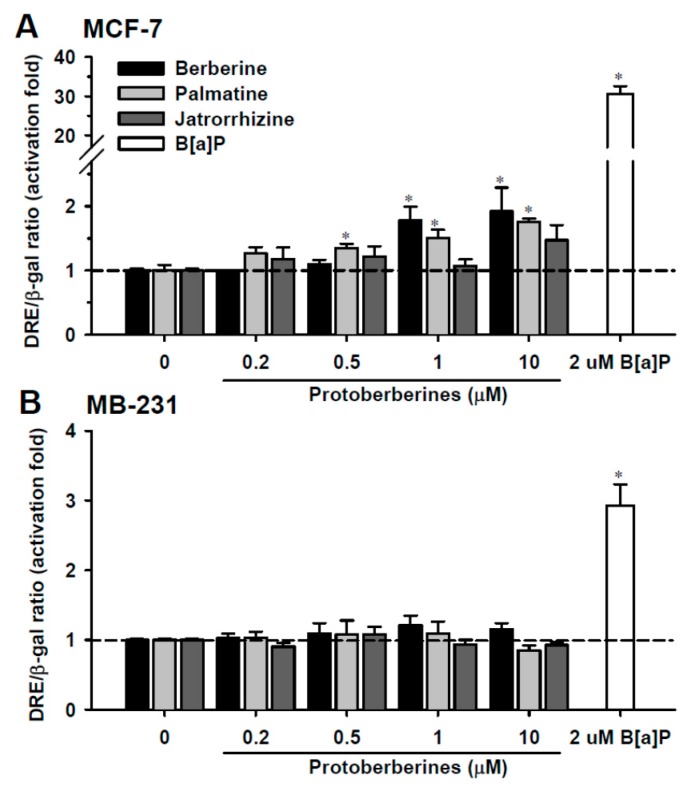
The dioxin-responsive element (DRE) activation by benzo(a)pyrene (B(a)P) and protoberberines in MCF-7 (**A**) and MB-231 (**B**) cells. After transfection of the DRE reporter construct, cells were exposed to B(a)P and protoberberines for 24 h. The DRE reporter luciferase activity was normalized by β-galactosidase activity. Data represent the mean ± SD of 3 separate experiments with 3 determinations for each experiment. * *p* < 0.05, compared with the control cells.

In MCF-7 cells, cell growth was significantly suppressed after 24-h exposure to cisplatin and doxorubicin at concentrations greater than 10 and 0.2 μM, respectively (Figure S1). The IC_50_ values for cisplatin and doxorubicin were 88.1 ± 7.5 and 1.8 ± 0.2 μM, respectively. These IC_50_ values were comparable to the reported values [19,20]. Cell viability was not affected by 24-h exposure to protoberberines at concentrations ≤10 μM (Figure S1). The cytotoxicity of berberine was increased after 48-h exposure. Berberine at >5 μM significantly decreased cell viability. In MB-231 cells, cell viability was not affected by 24-h exposure to protoberberines at concentrations ≤10 μM. Thus, the effects of 0.2–10 μM protoberberines on CYP1 were investigated. In MCF-7 and MB-231 cells, 24-h exposure to 2 μM B(a)P caused 31- and 3-fold activation of DRE-activated reporter expression, respectively (Figure 1A,B). In MCF-7 cells, berberine and palmatine, at respective exposure concentrations greater than 1 μM and 0.5 μM, caused a 1.4- to 2-fold activation of DRE, which was weaker than the activation induced by B(a)P (Figure 1A). Jatrorrhizine did not significantly activate the DRE-mediated induction. Results showed that in MCF-7 cells, the increased activation of reporter expression was in the order of berberine > palmatine > jatrorrhizine. In MB-231 cells, the reporter expression was not activated by any of the protoberberines (Figure 1B).

### 2.2. Effects of Berberine, Palmatine and Jatrorrhizine on mRNA Levels of CYP1s

**Figure 2:**
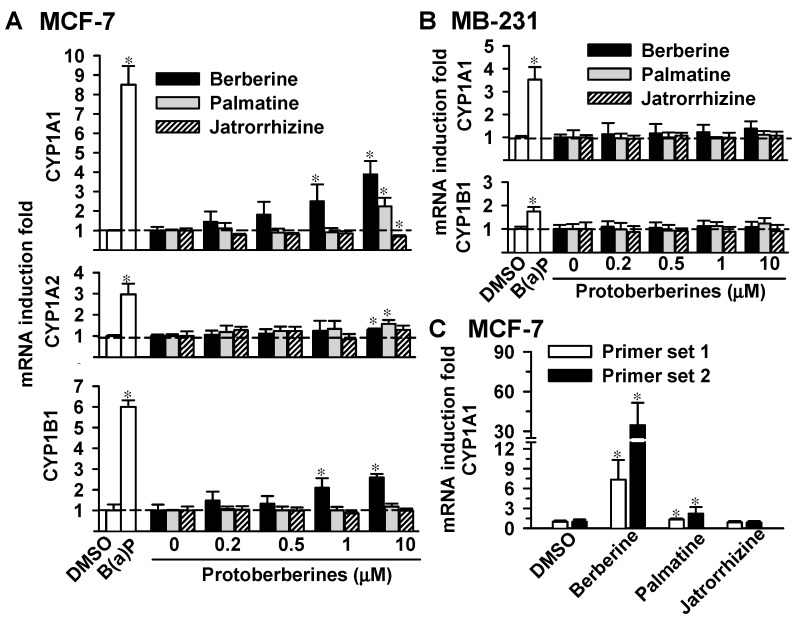
Effects of the protoberberines on the mRNA levels of CYP1A1, CYP1A2 and CYP1B1 in MCF-7 (**A**,**C**) and MB-231 (**B**) cells. Cells were exposed to B(a)P (2 μM) and protoberberines for 24 h. Total cellular RNA was isolated and subjected to reverse transcriptional reaction. In panels (**A**,**B**), the cDNA was subjected to PCR using the primer sets as described in the Materials and Methods. The PCR product was analyzed using gel electrophoresis and relative band intensity was determined. Results show the induction fold of the CYP1 mRNA level normalized by the mRNA level of β-actin. In panel (**C**), CYP1A1 mRNA level was determined using real-time PCR analysis and two primer sets were used as described in the Materials and Methods. Results show the induction fold of the CYP1A1 mRNA level normalized by the mRNA level of GAPDH. Data represent the mean ± SD of 3 separate experiments. * *p* < 0.05, compared with the control cells.

In MCF-7 cells, 24-h exposure to 2 μM B(a)P potently increased CYP1A1, CYP1A2 and CYP1B1 mRNA levels by 8-, 3- and 6-fold, respectively (Figure 2A). The 24-h exposure to berberine at 1 μM increased CYP1A1 and CYP1B1 mRNA levels by 3- and 2-fold, respectively. By increasing the exposure concentration to 10 μM, berberine significantly elevated the CYP1A1, CYP1A2 and CYP1B1 mRNA levels by 4-fold, 28% and 3-fold, respectively. Palmatine at 10 μM elevated CYP1A1 and CYP1A2 mRNA levels by 2-fold, whereas CYP1B1 mRNA level remained unchanged. Exposure to 10 μM jatrorrhizine decreased CYP1A1 mRNA levels by 32% without affecting CYP1A2 or CYP1B1. Among protoberberines, berberine caused the highest induction of CYP1A1 mRNA in MCF-7 cells. In MB-231 cells, the CYP1A2 mRNA levels were below detectable limits. The 24-h exposure to B(a)P (2 μM) caused 4- and 2-fold increases in the CYP1A1 and CYP1B1 mRNA levels, respectively. Berberine, palmatine and jatrorrhizine at a concentration up to 10 μM had no effects on CYP1A1 and CYP1B1 mRNA in MB-231 cells (Figure 2B). The differential induction of CYP1A1 mRNA by protoberberines in MCF-7 cells was further examined using real-time PCR analysis. By using two different primer sets, jatrorrhizine did not cause a significant change of CYP1A1 mRNA level. Berberine and palmatine at 10 μM induced CYP1A1 mRNA by 6–30 fold and 50%–227%, respectively (Figure 2C). Results of PCR analysis consistently demonstrated that berberine caused the most-potent induction of CYP1A1 mRNA.

### 2.3. Effects of Berberine on EROD Activity and the Protein Levels of CYP1A1 and CYP1B1

**Figure 3:**
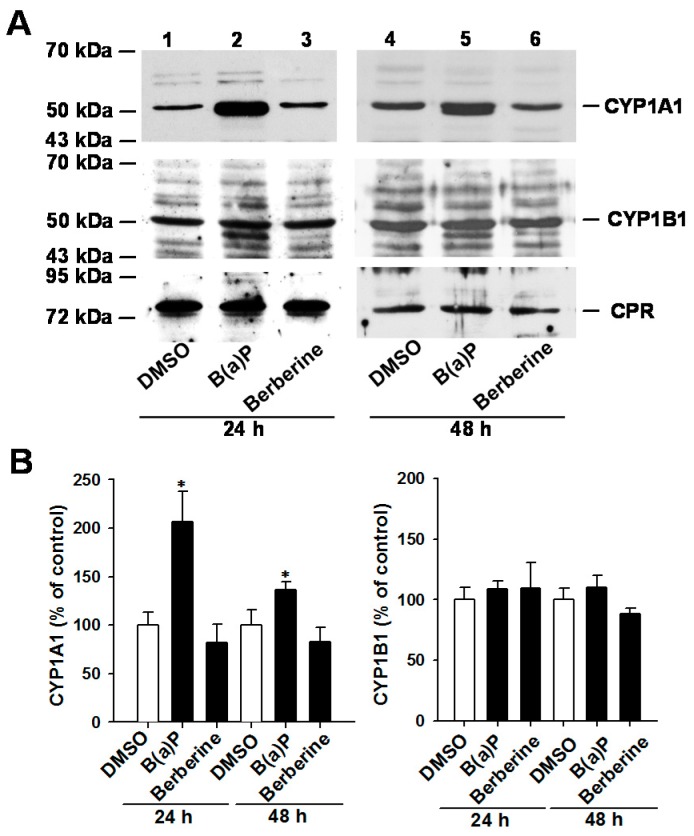
Immunoblot analyses of CYP1A1, CYP1B1 and CPR in the control, B(a)P (2 μM)- and berberine (10 μM)-treated MCF-7 cells. The vehicle control cells were exposed to the same amount of DMSO. After exposure to these chemicals, crude membrane fractions were prepared from cell lysate and subjected to electrophoresis. Immunoblot analysis was performed as described in the section of Methods. Panel (**A**) shows the representative blots; the band intensities of the blots were analyzed and the relative percentage to the control cells is shown in Panel (**B**). Results represent the mean ± SD of 3 separate experiments. * *p* < 0.05, compared with the control cells.

B(a)P (2 μM, 24 h) stimulated cellular EROD activity by 2-fold in MCF-7 cells, but not in MB-231 cells (Figure S2). In MCF-7 and MB-231 cells, the 24-h exposure to 0.2–20 μM berberine, palmatine and jatrorrhizine did not significantly affect cellular EROD activity. Even after prolonging the exposure time to 48 h, 10 μM berberine did not increase EROD activity in MCF-7 cells. Thus, the protein expression levels of CYP1A1 and CYP1B1 were further examined using immunoblot analysis. According to the reported gel mobilities of CYP1s and NADPH-P450 reductase (CPR) [21,22,23], the major immunoreacted protein band within the appropriate molecular weight range was determined. The apparent molecular weights of CYP1A1, CYP1B1 and CPR were estimated to be 53, 54 and 78 kDa, respectively. In MCF-7 cells, exposure to 2 μM B(a)P for 24 and 48 h increased CYP1A1 protein level by 2-fold and 36%, respectively (Figure 3A). These B(a)P exposure levels did not significantly change the CYP1B1 level (Figure 3B). Neither CYP1A1 nor CYP1B1 protein level was increased after 24 h and 48 h exposure to 10 μM berberine in MCF-7 cells. The CPR protein level remained unchanged after berberine exposure.

### 2.4. Effects of E2, Fulvestrant and ERα Knockdown on CYP1A1 mRNA Elevation by Berberine in MCF-7 Cells

**Figure 4:**
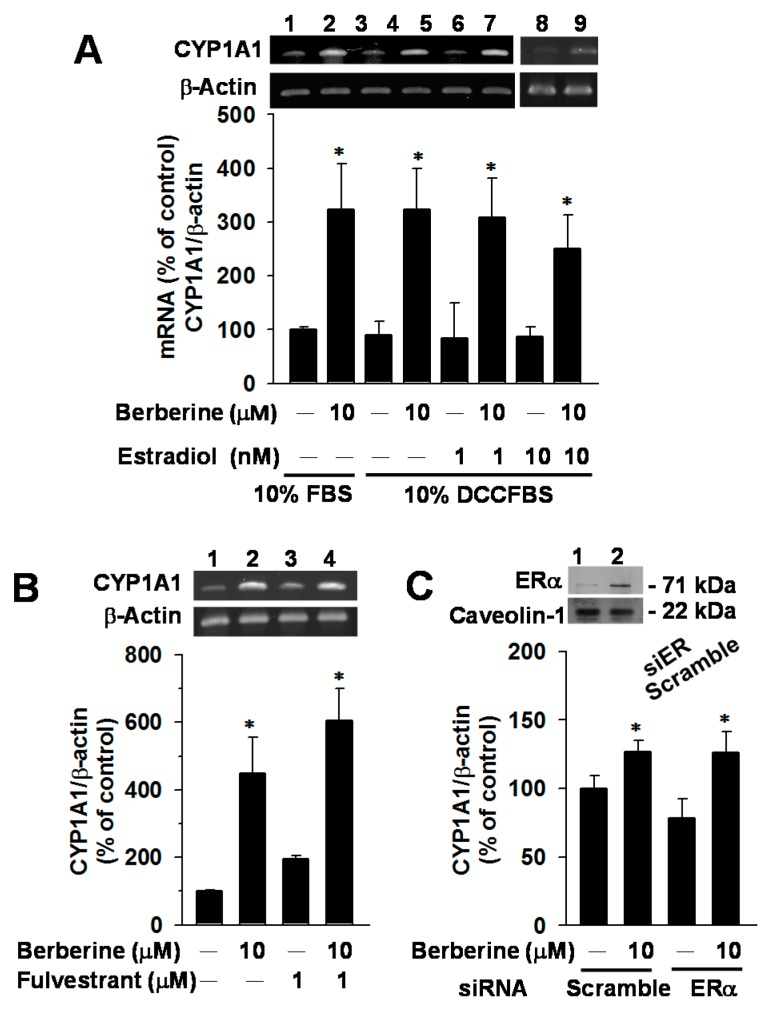
Effects of estradiol (**A**), fulvestrant (**B**) and ERα knockdown (**C**) on the mRNA levels of CYP1A1 and CYP1B1 in vehicle control and berberine-exposed MCF-7 cells. In panel (**A**), cells were cultured in the medium supplemented with 10% FBS or 10% DCCFBS, as indicated. The mRNA levels of CYP1A1 and β-actin were determined using RT–PCR analysis followed by gel electrophoresis. In panels (**B**,**C**), cells were exposed to berberine and/or fulvestrant in a 10% FBS-supplemented medium. In panel (**C**), cells were transfected with scramble and ERα siRNA in a serum-free medium. After 6 h, the medium was replaced by a FBS-supplemented medium. Cells were cultured in the incubator for 18 h and then exposed to berberine for 24 h. Cell lysates were prepared and subjected to the immunoblot analysis as described in the Materials and Methods. The level of caveolin-1 was determined as the control for loading equal amount of protein of lysate. The representative blot is shown on the top. Data represent the mean ± SD of 3 separate experiments with 4 determinations in each experiment. * *p* < 0.05, compared with the cells without berberine-treatment.

To examine the interference of ERα signaling, effects of E2 (1 and 10 nM), fulvestrant (ERα antagonist) (1 μM) and ERα knockdown were studied (Figure 4). In cells cultured in DCC-stripped FBS (DCCFBS)-supplemented medium, E2 alone did not change CYP1A1 mRNA level. The berberine-mediated increase in CYP1A1 mRNA was not affected by concurrent exposure to E2 at a concentration up to 10 nM (Figure 4A). In cells cultured in FBS-supplemented medium, the ER antagonist fulvestrant had no influence on the berberine-mediated CYP1A1 mRNA induction (Figure 4B). The transfection of siERα resulted in a 70% decrease of basal ERα expression, but did not diminish the berberine-mediated CYP1A1 mRNA induction (Figure 4C). Results revealed that berberine-mediated CYP1A1 mRNA induction could not be affected by the ERα signaling.

### 2.5. The Involvement of miR-21-3p in the Post-Transcriptional Regulation of CYP1A1 by Berberine

**Figure 5:**
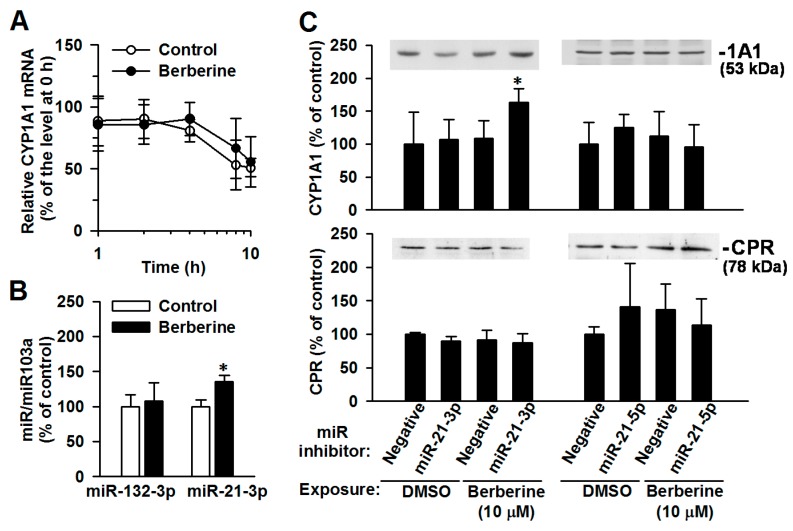
Effects of berberine on the CYP1A1 mRNA degradation (**A**) and miR levels (**B**), and the effects of miR inhibitors on CYP1A1 protein levels (**C**) in control and berberine-treated MCF-7 cells. Cells were exposed to vehicle (control) and berberine (10 μM) for 24 h. (**A**) Cells were exposed to actinomycin for 1, 2, 4 and 8 h and then total RNA was isolated. CYP1A1 mRNA level was determined using RT-PCR analysis followed by gel electrophoresis. Results represent the mean ± SD of 3 experiments. (**B**) The miR levels were determined using real-time PCR analysis. Results represent the mean ± SD of 4 separate experiments. * *p* < 0.05, compared with control cells. (**C**) After transfection with miR inhibitors for 48 h, cells were exposed to berberine for 24 h. Cell lysates were prepared and subjected to immunoblot analysis of CYP1A1 and CPR. The representative blots are shown on the top of the results of relative band-intensity determination. The band intensity of the DMSO-treated cells with the negative transfection was considered as 100%. Results represent the mean ± SD of 3 separate experiments. * *p* < 0.05, compared with respective negative transfection control with the same chemical exposure.

To illustrate post-transcriptional regulation, the stability of CYP1A1 mRNA was first determined. The berberine (10 μM, 24 h)-mediated change to the half-life of CYP1A1 mRNA was minimal (control cells: 9.3 ± 3.4 h; berberine-exposed cells: 11.7 ± 2.4 h) (Figure 5A). Berberine exposure significantly elevated the miR-21-3p level by 36 ± 18%, without affecting the miR-132-3p level (Figure 5B). The miR-142-3p level was below detectable limits in both the control and berberine-exposed cells. Transfection of a miR-21-3p hairpin inhibitor restored the berberine-mediated induction of CYP1A1 protein with a 50 ± 19% increase (Figure 5C). In miR-21-5p hairpin inhibitor-transfected cells, CYP1A1 protein level remained unchanged after berberine exposure. The transfection of miR inhibitors did not affect the expression level of the CYP electron-transfer partner, CPR [2]. These results revealed that berberine stimulated miR-21-3p, suppressing the elevation of CYP1A1 protein.

## 3. Discussion

In MCF-7 cells, our findings revealed that protoberberines did not cause cytotoxicity at concentrations below 10 μM. At a concentration of ≤1 μM, berberine and palmatine activated DRE-mediated reporter expression in MCF-7 cells, while jatrorrhizine did not. Among protoberberines, berberine caused the most-potent AhR activation and CYP1A1 mRNA stimulation in MCF-7 cells, but not in MB-231 cells. Consistent with the report of the effect of TCDD [24], the increases in CYP1B1 mRNA by berberine and palmatine were less than the elevation of CYP1A1 mRNA. The methylenedioxyl moiety can act as a crucial substitute on the D-ring of a protoberberine for AhR activation. The IC_50_ values for the inhibition of recombinant human CYP1A1 were in the following order: berberine (1.38 μM) < jatrorrhizine (2.17 μM) < palmatine (8.71 μM) [12]. Although berberine was predicted to potentially activate AhR through inhibition of CYP1A1 in cells [13], the CYP1A1 inhibitory effects of these protoberberines did not correlate with their respective effects on AhR activation in this report. In addition to the ligand binding affinity, the differential DRE activation by protoberberines can be attributed to factors including bioavailability and the metabolic process. On the other hand, the increase in the mRNA of hepatic CYP1 isoforms from berberine was not only found in the cultured cells [14], but also in mice in vivo [16]. Further in vivo study is warranted to assess the effect of berberine on breast AhR activation.

In rats, B(a)P elevated the CYP1A1 mRNA level in the ovaries by an amount comparable to that in the liver [24]. However, the CYP1A1 protein level and microsomal EROD activity were induced by B(a)P in the liver, but not the ovaries. B(a)P induced ovary miR-21 but decreased hepatic miR-21. Elevated levels of miRs, including miR-21, can contribute to the post-transcriptional downregulation of ovary CYP1A1 protein expression. Although one immunoblot showed a weak to mild elevation in CYP1A1 protein in MCF-7 cells exposed to 10–40 μM berberine for 48 h [25], the statistical significance has not been proven. Our findings revealed that neither CYP1A1/1B1 protein levels nor EROD activity in MCF-7 cells were significantly affected by protoberberines at a non-cytotoxic concentration (≤10 μM). This finding was consistent with that of a mouse study [16] that showed that liver microsomal EROD activity was unaffected by berberine when CYP1A2 mRNA was elevated. Unlike the differential miR-21 induction by B(a)P in rat liver and ovaries [24], the induction of miR-21-3p occurred in both hepatoma HepG2 [18] and breast cancer MCF-7 cell lines. CYP1A1 protein level was significantly induced by berberine in MCF-7 cells after the transfection of a miR-21-3p hairpin inhibitor, demonstrating that miR-21-3p suppressed CYP1A1 protein induction. While berberine increased CYP1A1 mRNA to a level approaching that of TCDD-exposed HepG2 cells [14], our findings suggested that an increase in miR-21-3p [20] might be the reason for the mild increase in CYP1A1 protein level by berberine as compared to that induced by TCDD. The overexpression of miR-21-3p ameliorated the nuclear factor (NF)-κB signaling induced by IL-1β [26]. The association of berberine-mediated AhR activation with the miR-mediated inflammatory modulation by berberine can be crucial in future studies.

In conclusion, at a non-cytotoxic and pharmacologically achievable concentration [27], berberine and palmatine activated AhR, which has been reported to be involved in the modulation of the xenobiotic metabolism and inflammatory response [1,28]. While the CYP1A1 mRNA was increased, berberine-induced miR-21-3p suppressed the increase in CYP1A1 protein and function. Our findings warrant future investigation of the contribution of AhR activation and miR-21-3p to the pharmacological activity of berberine in vivo.

## 4. Materials and Methods

### 4.1. Chemicals, Enzymes and Antibodies

Dulbecco’s Modified Eagle’s Medium (DMEM, Gibco Cell Culture Media) and peroxidase conjugated rabbit anti-mouse IgG (31450) and goat anti-rabbit IgG (H + L) (31460) were purchased from Thermo Fischer Sci. Inc. (Waltham, MA, USA). Fetal bovine serum (FBS) was purchased from Biological Industries (Kibbutz Beit HaeMek, Israel). Glutamine, the non-essential amino acid and the mixture of antibiotics (penicillin, streptomycin and amphotericin) were purchased from Caisson Laboratories Inc. (North Logan, UT, USA). Dimethyl sulfoxide (DMSO), glycerol, methanol and triton X-100 were purchased from Merck KGaA (Darmstadt, Germany). Berberine chloride, cisplatin, dextran-coated charcoal (charcoal/dextran, DCC, C6241), doxorubicin hydrochloride, E2, 7-ethoxyresorufin and 3-(4,5-dimethyl-thiazol-2yl)-2,5-diphenyl tetrazolium bromide (MTT) were purchased from Sigma-Aldrich (St. Louis, MO, USA). Hairpin inhibitors of miR-21-3p and miR-21-5p were purchased from Thermo Fischer Sci. Inc. (Biosciences, Lafayette, CO, USA). Palmatine and jatrorrhizine were isolated and purified from *Fibraurea tinctoria* as previously described [19]. Rabbit polyclonal antibody against human CYP1A1 (AB10328), which immunoreacted with CYP1A1 in a human thyroid cancer cell line, was purchased from Merck Millipore (Billerica, MA, USA) [22]. The monoclonal antibody against the oligopeptide fragment of human CYP1B1 (G-4, sc-374228), which immunoreacted with CYP1B1 in human hepatoma cells [23], was purchased from Santa Cruz Biotech., Inc. (Dallas, TX, USA). Rabbit polyclonal antibody against rat NADPH-P450 reductase (CPR) (Ab13513), which immunoreacted with human CPR [21], was purchased from Abcam (Cambridge, UK). Rabbit anti-human ERα (PLA0113), which immunoreacted with ERα in MCF-7 cells [29] was purchased from Sigma Aldrich (St. Louis, MO, USA). Rabbit polyclonal anti-caveolin (610059), which immunoreacted with human, mouse and rat caveolin, was purchased from BD Biosciences Pharmingen (Franklin Lakes, NJ, USA).

### 4.2. Cell Culture and Exposure

MCF-7 and MB-231 cells were cultured in DMEM medium supplemented with 10% FBS, 1% l-glutamine, 1% mixture of antibiotics and 1% non-essential amino acid. After seeding, cells were cultured for 24 h before exposure to benzo(a)pyrene and protoberberines (in DMSO). In the study of the influence of E2 (in methanol), DCCFBS was prepared following the instructions on the use of DCC and about 97% of E2 was removed, monitored using the E2 EIA kit (Cayman Chemical Company, MI, USA). Before protoberberine- and E2-exposure, cells were cultured for 24 h in 10% DCCFBS-supplemented medium. The final concentration of vehicle in the medium was 0.1%. Cell viability was monitored by MTT assay, as previously described [30].

### 4.3. DRE Reporter Assay

The DRE-luciferase reporter construct was prepared as described previously [31]. After seeding (7.5 × 10^4^ cells/well, 24-well plate) and overnight culture, MCF-7 cells were transiently transfected with the reporter vector and the β-galactosidase control vector using lipofectamine 2000 reagent (Invitrogen Co., Carlsbad, CA, USA). After incubation for 6 h and following exposure to B(a)P and protoberberines for 24 h, cell lysate was prepared using the reporter lysis buffer (Progema, Madison, WI, USA), and luciferase and β-galactosidase activities of the cell lysate were determined [31]. The activation of DRE was defined as the relative luminescence unit/absorbance at 420 nm.

### 4.4. Immunoblot Analysis

In the immunoblot analysis of CYP1s, crude membrane fractions of cell lysate were prepared as previously described [32]. Crude membranes (50 μg protein/well) were resolved by electrophoresis on a 7.5% or 10% (*w*/*v*) polyacrylamide gel and electrotransferred from the slab gel to a nitrocellulose membrane. Prestained molecular weight markers, Sharp Protein Markers II and III, were purchased from Yeastern Biotech Co., Ltd. (Taipei, Taiwan, ROC). After incubation with 5% non-fat milk in phosphate-buffered saline (PBS) at 37 °C for 1 h, the membranes were incubated with anti-CYP1A1 (1:2500), anti-CYP1B1 (1:200) and anti-CPR (1:2000) in PBS containing 1% non-fat milk overnight at 4 °C. In the immunoblot analysis of ERα and caveolin-1, cell lysate was prepared as described previously [29] and subjected to electrophoresis as described above. The nitrocellulose membrane was incubated with anti-ERα (1:2000) and anti-caveolin-1 (1:2000) in non-fat milk-containing PBS. After four washes, the immunoreactive proteins were detected using horseradish peroxidase-conjugated IgG (1:1000 or 1:2000) and an enhanced chemiluminescence detection kit (PerkinElmer Life and Analytical Sciences, Inc., Shelton, CA, USA). Relative band intensity was analyzed using the Multi Gauge software (Ver. 2.2, FUJIFILM Co., Tokyo, Japan).

### 4.5. Determination of Cellular EROD Activity

EROD activity was determined using the methods of Peters et al. [33], with mild modifications. 4 × 10^5^ MCF-7/1 × 10^5^ MB-231 cells were seeded in each well of a 24-well plate. After B(a)P and protoberberine treatments, cells were rinsed twice with PBS and then incubated for 3 h and at 37 °C in a serum-free medium containing 5 mM MgCl_2_, 5 μM 7-ethoxyresorufin and 10 μM dicumarol. The difference in fluorescence intensity before and after incubation was determined and the difference was normalized by protein concentration (Protein assay kit I, Bio-Rad Laboratories, Inc., Richmond, CA, USA).

### 4.6. Stability and Reverse Transcription (RT)-Polymerase Chain Reaction (PCR) Analysis of mRNA

To determine the mRNA stability, cells were exposed to 5 μg/mL actinomycin 0, 1, 2, 4 and 8 h prior to RNA isolation. Total cellular RNA was isolated using TRIzol reagent (Invitrogen Co., Carlsbad, CA, USA) following the manufacturer’s instructions. RT step occurred at 42 °C for 60 min and then at 95 °C for 5 min. The resulting cDNA was subjected to PCR amplification using primer sets for CYP1A2*1 (NM_000761.4) [34], CYP1B1*1 (NM_000104.3) [6] and β-actin (NM_001101) [35]. For CYP1A1*1 (NM_000499.4), primer set 1: forward primer 5′-TAGACACTGATCTGGCTGCAG-3′ and the reverse primer 5′-GGGAAGGCTCCATCAGCATC-3′ was used. The amplification of CYP1A1, CYP1A2, CYP1B1 and β-actin was carried out for 35, 40, 35 and 25 cycles, respectively. The PCR products were analyzed by electrophoresis on 1.5% agarose gels and DNA was visualized via ethidium bromide staining. Band intensity was analyzed using AlphaImager 2200 (Alpha Innotech Co., San Leandro, CA, USA). In the real-time PCR analysis of CYP1A1 mRNA level, both primer set 1 and a reported primer set 2 [23] were used to further examine the differential effects of protoberberines. The cDNA (1 μL RT product) was subjected to PCR amplification with an initial enzyme activation step of 95 °C for 2 min followed by 45 cycles each at 95 °C for 5 s, 60 °C for 10 s and 72 °C for 10 s. The primer set for glyceraldehyde 3-phosphate dehydrogenase (GAPDH) (NM_002046.5) was forward, CGGAGTCAACGGATTTGGTCGTAT; reverse, AGCCTTCTCCATGGTGGTGAAGAC. A Bioline FAST SYBR No-ROX-mix kit (Bioline Reagents, London, UK) was used and a fractional PCR threshold cycle number (*C*_t_ value) was determined using LightCycler 480II (Software version 1.5.0.39; Roche Diagnostics GmbH, Mannheim, Germany). The relative mRNA expression level was normalized to the GAPDH transcript level, which allowed the target cDNA calculation by 2^−(Ct *CYP1A1*−Ct *GAPDH*)^.

### 4.7. Transfection of Estrogen Receptor siRNA

After seeding (5 × 10^4^ cells/well in a 6-well plate), MCF-7 cells were cultured for 24 h and then transfected with ERα siRNA (50 nM) and scrambled siRNA (40 nM) using lipofectamine 2000 (Invitrogen, Carlsbad, CA, USA) following the manufacturer’s instructions. Human ER siRNA (s4824) and scrambled siRNA were purchased from Ambion (Life Technologies, Thermo Fisher Scientific). The expression level of ERα protein was determined using immunoblotting analysis as described above to ensure the knockdown of ERα protein [29].

### 4.8. Determination of miRs and Transfection of the miR Inhibitors

After exposing MCF-7 cells to 10 μM berberine for 24 h, miRs were isolated from cells using a miRCURY™ RNA isolation kit (Exiqon A/S, Vedbaek, Denmark). Following the manufacturer’s instruction, the miR levels were determined using reverse transcription (miRCURY LNA™ Universal RT microRNA PCR, Exiqon A/S, Denmark) followed by real-time PCR (miRCURY LNATM Universal RT microRNA PCR, Exiqon A/S, Denmark) and analyzed using LightCycler 480 II/96 (Roche Diagnostics Ltd., Rotkeruz, Switzerland). Transfection of the miR inhibitors and negative control was performed using Thermo Scientific Dharma FECT transfection reagents, as per the instructions manual. Cell lysate was prepared for the immunoblot analysis as described above.

### 4.9. Statistical Analysis

The difference in experiments with more than two sets of data from cells exposed to increasing concentrations of protoberberines was determined using one-way analysis of variance (ANOVA), followed by Dunnett’s test (SigmaStat version 2.03, SPSS Inc., Chicago, IL, USA). The significant difference between the control and each chemical-treated group was determined using Student’s *t*-test; *p* < 0.05 was considered statistically significant.

## Supplementary Materials

The following are available online. Figure S1: Effects of the protoberberines berberine, palmatine and jatrorrhizine on viability of MCF-7 and MDA-MB-231 (MB-231) cells, Figure S2: Effects of protoberberines on the 7-ethoxyresorufin *O*-deethylation (EROD) activity in MCF-7 and MDA-MB-231 (MB-231) cells.
