# Supplementary material for: Berberine Activates Aryl Hydrocarbon Receptor but Suppresses CYP1A1 Induction through miR-21-3p Stimulation in MCF-7 Breast Cancer Cells
Source: Molecules. 2017 Oct 28;22(11):1847. doi: 10.3390/molecules22111847 (PMC6150360; doi:10.3390/molecules22111847)
Supplement: Supplementary file 1 [file molecules-22-01847-s001.pdf]

Supplementary Data

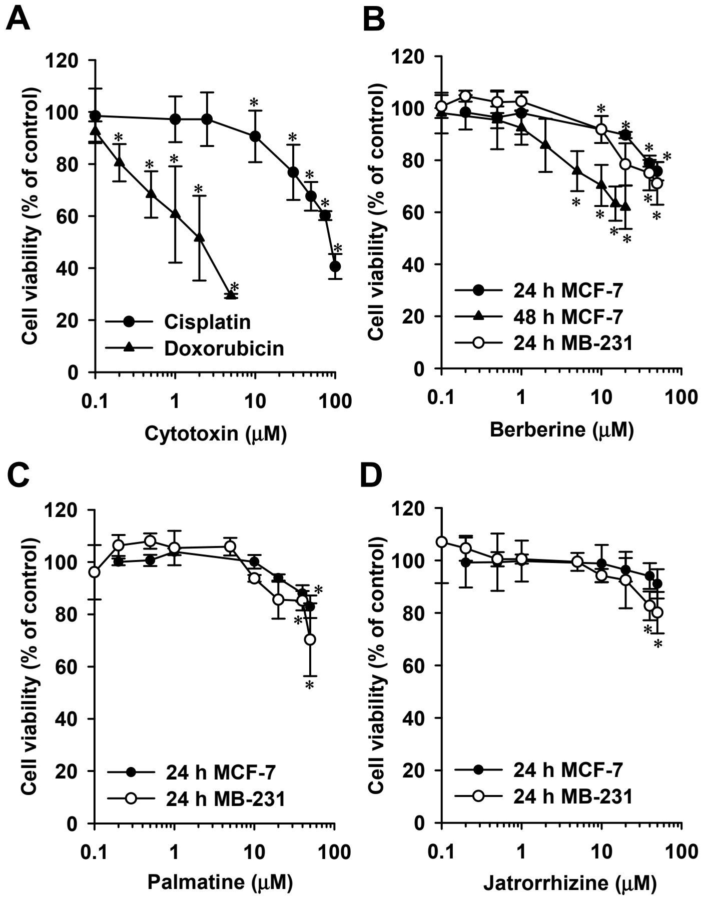

Figure S1. Effects of the protoberberines berberine, palmatine and jatrorrhizine on viability of MCF-7 and MDA-MB-231 (MB-231) cells. In panel (**A**), MCF-7 cells were exposed to cisplatin and doxorubicin for 24 h and cell viability was monitored using MTT assay. Data represent the mean ± SD of 3 separate experiments, with 3-4 determinations in each experiment. * $p < 0.05$, compared with the control cells. In panels (**B**)–(**D**), cells were exposed to protoberberines at the concentrations indicated for 24 h or 48 h. Results show the relative cell viability compared to control cells exposed to the same concentration of DMSO. Data represent the mean ± SD of 5 and 3 separate experiments, with 4 determinations for each experiment, for MCF-7 and MB-231 cells, respectively. * $p < 0.05$, compared with the control cells.

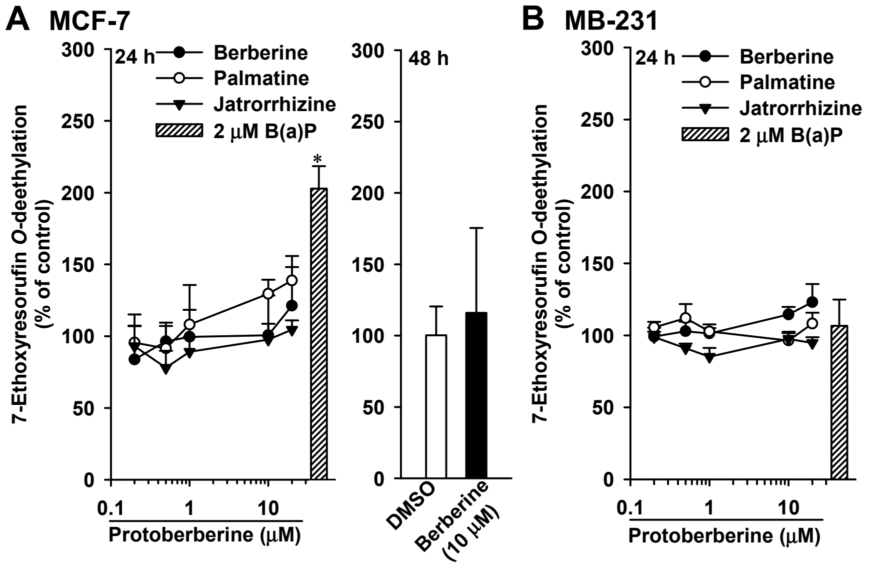

Figure S2. Effects of protoberberines on the 7-ethoxyresorufin *O*-deethylation (EROD) activity in MCF-7 and MDA-MB-231 (MB-231) cells. The basal EROD activity in MCF-7 and MB-231 cells were 0.22 ± 0.08 and 0.17 ± 0.08 pmol/min/mg protein, respectively. Results represent the mean ± SD of 3 and 4 experiments with duplicated determinations for MCF-7 and MB-231 cells, respectively. * $p < 0.05$, compared with the control cells.
